# Cauda equina neuroendocrine tumour of the filum terminale: a rare cause of persistent sciatica

**DOI:** 10.1093/omcr/omaf179

**Published:** 2025-09-28

**Authors:** Theoklis Kouyialis, David Halliday, Christophoros Christophorou, Christina Oxinou

**Affiliations:** College of Medicine and Veterinary Medicine, The University of Edinburgh, 49 Little France Cres, Edinburgh EH16 4SB, United Kingdom; Department of Neurosurgery, Aretaeio Private Hospital, 55-57, Andrea Avraamidi st, Strovolos 2024, Cyprus; College of Medicine and Veterinary Medicine, The University of Edinburgh, 49 Little France Cres, Edinburgh EH16 4SB, United Kingdom; Department of Neurosurgery, Aretaeio Private Hospital, 55-57, Andrea Avraamidi st, Strovolos 2024, Cyprus; Histopathology, Oxinou Diagnostic Pathology Laboratory, 1 Arch. Makariou III Av., Nicosia, Cyprus

**Keywords:** neurology, oncology

## Abstract

Cauda equina neuroendocrine tumours (CENETs), previously known as spinal paragangliomas (PGLs), are an extremely rare subset of spinal tumours that involve the cauda equina and filum terminale. They are classified as WHO Grade 1 and, thus, are largely benign. Their rarity, along with the overlap of their symptoms with more common spinal pathologies, leads to their frequent misdiagnosis. We present the case of a man in his mid-50s with a history of LBP exacerbated at night and right-sided sciatica with an L4 radicular distribution. He was referred to our team after being misdiagnosed and treated for lumbar disc herniation. A contrast-enhanced MRI revealed the presence of a large intradural lesion at the level of L4, treated by gross total resection (GTR) and later identified as a CENET. This case aims to enhance clinicians’ understanding of CENETs, thereby preventing future misdiagnoses and improving patient outcomes.

## Introduction

Cauda Equina Neuroendocrine Tumours (CENETs), previously known as Spinal Paragangliomas (PGLs), are an extremely rare subset of highly vascularised [1–4], intradural and extramedullary [1, 3–5] tumours involving the cauda equina and filum terminale. They are classified as WHO Grade 1 [1–5] and thus are largely benign [1, 3, 6]. Common symptoms are low back pain (LBP), sciatica and motor deficits in the lower limbs. CENETs occur predominantly in men in their 40s [1–3, 5] and are sporadic, with no known hereditary component [1, 5, 6]. Their lack of symptom specificity and similarity to other spinal tumours on imaging make diagnosis challenging [1, 3, 5].

We report the case of a man in his 50s who presented to our team with right-sided sciatica and low back pain at night after previously being diagnosed with and treated for a lumbar disc herniation. An intradural lesion was discovered on contrast-enhanced MRI, and he underwent Gross Total Resection (GTR), after which he made a full and uneventful recovery. The lesion was histologically identified as a CENET.

## Case report

We present the case of a male patient in his mid-50s. He had twice previously presented to a different centre after experiencing persistent right leg sciatica (visual analogue scale (VAS) 6–7/10), where he was diagnosed with a lumbar disc herniation and treated with non-steroidal anti-inflammatories (NSAIDs) and corticosteroids on both occasions. This treatment provided moderate symptom relief, allowing him to return to normal activities with only some discomfort, with his symptoms fully recurring around 4 months later in each case. He sought a second opinion after his second recurrence, presenting to our team with LBP and right leg sciatica (VAS 10/10), which would wake him at night but without sphincter involvement or motor/sensory dysfunction.

**Figure 1:**
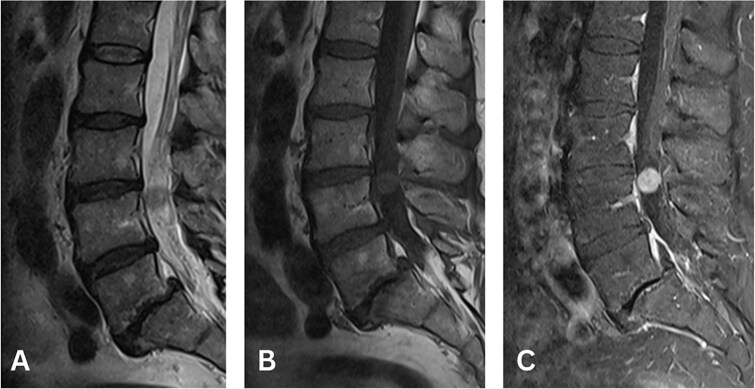
Magnetic resonance imaging (MRI) of the lumbar spine sagittal sequences revealing a round, intradural, extramedullary lesion at the level of the superior endplate of the L4 vertebral body which (A) on T2-weighted imaging (T2WI) demonstrates heterogeneous iso to hyperintense signal, (B) on T1WI demonstrates isointense signal, and (C) on post-contrast T1WI with fat suppression demonstrates homogenous diffuse enhancement.

Magnetic resonance imaging (MRI) revealed the presence of an intradural lesion near the superior border of the L4 vertebral body, with heterogeneous iso to hyperintensity on T2, isointensity on T1 and homogenous diffuse enhancement on T1 with contrast and fat suppression (Fig. 1). Our differential diagnoses for the lesion included ependymoma, meningioma or schwannoma.

A GTR of the tumour was performed through a partial L3-L4 laminectomy. The dura was opened, and the dural opening was stabilised with Prolene sutures. The arachnoid was then opened, and the nerve rootlets were identified and mobilised. The upper and lower edges of the tumour were secured with surgical patties, and the tumour was identified to arise from and involve the filum terminale. This was confirmed with intraoperative neurophysiology to avoid damage to the neural structures. The nerve roots were detached from the tumour, facilitating en-block removal of the tumour along with the filum, followed by coagulation and the addition of a local haemostatic agent. The dura was sutured with 6.0 Prolene after meticulous haemostasis, and a Valsalva manoeuvre confirmed the absence of a CSF leak. The patient made a full and uneventful recovery.

**Figure 2:**
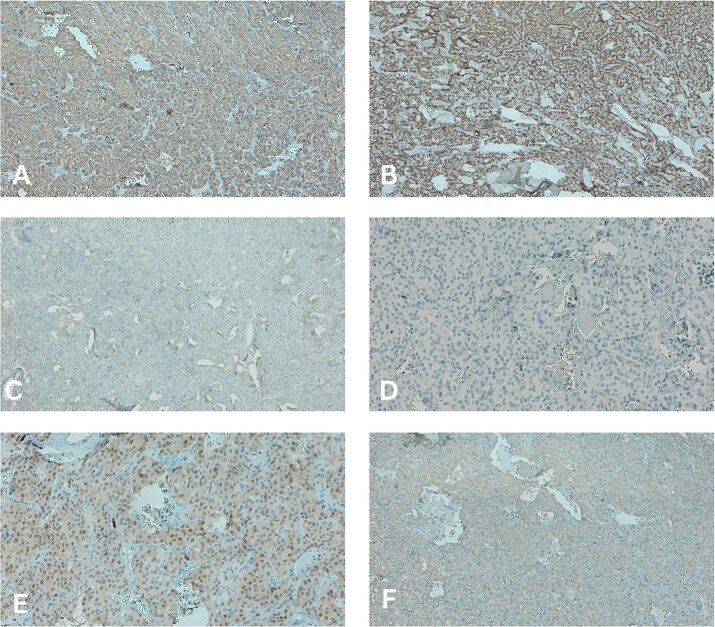
Immunohistochemical staining showing (A) chromogranin a positivity, (B) cytokeratin AE1/AE3 positivity, (C) epithelial membrane antigen negativity, (D) glial fibrillary acidic protein negativity, (E) soluble 100 protein positivity and (F) Synaptophysin positivity.

Histopathological examination of the lesion revealed delicate encapsulation with chief cell nests (Zellballen structures) surrounded by sustentacular cells, staining positive for Soluble 100 (S100) protein. Immunohistochemical testing was performed, revealing the neoplasm was positive for Chromatogranin A, Synaptophysin and Cytokeratin AE1/AE3 and negative for Epithelial Membrane Antigen (EMA) and Glial Fibrillary Acidic Protein (GFAP) (Fig. 2). A low KI-67 proliferative marker also confirmed that the tumour was benign. The above ruled out an ependymoma and produced the final diagnosis of a CENET.

## Discussion

The reclassification of CENETs by the WHO in 2022 is owed to a multitude of newly published evidence. Though they were previously classified as spinal PGLs due to their expression of chromogranin A and synaptophysin [1, 3–5], they were found to express cytokeratins, not seen in extra-spinal PGLs, as well as different transcription factors [5, 6]. Further evidence was provided by the fact that they occur sporadically rather than having a pattern of inheritance like PGLs and by differences in chromosomal copy number and epigenetics [5, 6].

The very small number of reported cases (300 since the 1970s [1, 2]) makes identification challenging. Symptoms are non-specific, commonly LBP and sciatica, [1–5] followed by lower limb deficits. Bowel and bladder dysfunction have been reported in rare cases [2–5].

On MRI, CENETs commonly present as hypo-isointense on T1, homogeneously hyperintense on T2 and homogeneously enhanced on T1 with contrast [1, 3–5]. They have also been described as having a peripheral hypointense rim on T2 related to hemosiderin deposits [1, 3, 5] and serpiginous flow voids, suggesting hypervascularity [1]. Yang et al have noted a ‘salt and pepper’ appearance due to rich vascularisation that may help differentiate CENETs from other spinal tumours [3]. Yi et al have also shown potential for the use of the ‘Tadpole’ sign, whereby the tumour is the head of the tadpole, and the dilated, tortuous vessels above or below it are the tail of the tadpole [7]. Finally, CENETs can be differentiated from ependymomas by their contrast enhancement, which is heterogeneous rather than homogeneous in ependymoma [1]. These findings alone, however, are not sufficient to distinguish them from more common spinal tumours, and they are often misdiagnosed as ependymomas, meningiomas, hemangiomas, or schwannomas [1–3, 5].

Due to the lack of specificity in both clinical and imaging findings, histopathology and immunohistochemistry are important in confirming a diagnosis of CENET. Typical appearance includes a ‘Zellballen’ structure of chief cells with round to elongated and uniform Nuclei [8]*.* They also show S100 positivity, typically in the surrounding sustentacular cells, chromogranin positivity, and cytokeratin positivity [8]. They can be differentiated from ependymomas by their EMA negativity, and from schwannomas or other gliomas by their GFAP negativity [2], as was seen in our case.

GTR is considered the gold standard treatment, providing a much lower risk of recurrence (5%) compared to STR (66.7%) [1]. Radiotherapy has been suggested in cases of Sub-Total Resection (STR) [3], but more evidence would be required to recommend its use.

To provide a better understanding of the common symptoms of this rare neoplasm, we conducted a literature review of past reported cases of CENET, using the search term “(“cauda equina” OR “spine” OR “lumbar”) AND (“paraganglioma” OR “neuroendocrine tumour”)”. All non-review articles in English published between 2000 and 2025 that describe patient symptoms resulting from a primary paraganglioma or CENET in the lumbar or sacral region were included, totalling 67 articles [1–5, 7, 9–69], of which 19 were case series and 48 were isolated case reports. From these articles, we extracted patient demographics, symptoms, and symptom duration for 194 patients (Table 1) and recurrence status for 144 patients (not shown). The most typical symptoms were LBP and lower extremity pain (78% and 65% respectively). Our findings highlighted a rare manifestation of CENET, which occurred in 5 individuals (2.6%), with the only indications being papilledema and symptoms of raised ICP (headache, nausea). Finally, of 144 patients with available recurrence data, only 6 (4%) experienced recurrences.

**Table 1:** Results of literature search showing patients diagnosed with CENET between 2000 and 2025, with key demographic characteristics and common symptoms.

Patient characteristic	Summary statistic (N = 194)
Age (Mean ± SD) (years)	49.1 ± 13.7
Sex (%) Female Male Symptoms (%) Low back pain Lower extremity pain Paraesthesia/Dysesthesia Weakness Bowel/Bladder dysfunction Papilledema	36 64 78 65 27 22 14 2.6
Symptom duration (Median (Q1-Q3)) (months)	10 (4–24)

Our case highlights the need for a higher index of suspicion for serious causes of low back pain and sciatica in patients with repeated and rapid recurrence of symptoms after treatment. Though the patient was twice misdiagnosed and his symptoms worsened each time he recurred, imaging was not obtained in either case, leading to the continuation of incorrect treatment. The symptom of nocturnal pain occurred late in our patient, but should always be treated as a warning sign of possible neoplasm and investigated diligently.

Though the presentation of CENET is typical of more common spinal pathologies, such as lumbar disc protrusion, a lack of response to treatment should prompt rapid radiological investigation to guide further management. Although our patient made a full recovery, long-term neurological impairment has been reported in some patients [3], especially when the correct diagnosis is delayed, and recovery can be long, difficult and costly despite GTR.
